# Pediatric idiopathic steroid-sensitive nephrotic syndrome: diagnosis and therapy —short version of the updated German best practice guideline (S2e) — AWMF register no. 166-001, 6/2020

**DOI:** 10.1007/s00467-021-05135-3

**Published:** 2021-06-06

**Authors:** Rasmus Ehren, Marcus R. Benz, Paul T. Brinkkötter, Jörg Dötsch, Wolfgang R. Eberl, Jutta Gellermann, Peter F. Hoyer, Isabelle Jordans, Clemens Kamrath, Markus J. Kemper, Kay Latta, Dominik Müller, Jun Oh, Burkhard Tönshoff, Stefanie Weber, Lutz T. Weber, on behalf of the German Society for Pediatric Nephrology

**Affiliations:** 1 Faculty of Medicine and University Hospital Cologne, Pediatric Nephrology, Children’s and Adolescents’ Hospital, University of Cologne, Cologne, Germany; 2 Department II of Internal Medicine and Center for Molecular Medicine Cologne (CMMC), Faculty of Medicine and University Hospital Cologne, University of Cologne, Cologne, Germany; 3 Cologne Cluster of Excellence on Cellular Stress Responses in Ageing-Associated Diseases (CECAD), Cologne, Germany; 4 Department of Pediatrics, Städtisches Klinikum Braunschweig, Braunschweig, Germany; 5 Pediatric Nephrology, Charité Children’s Hospital, Berlin, Germany; 6 Center for Children and Adolescents, Pediatric Clinic II, University of Duisburg-Essen, Essen, Germany; 7 Bundesverband Niere eV (German National Kidney-Patients Association), Mainz, Germany; 8 Division of Pediatric Endocrinology & Diabetology, Center of Child and Adolescent Medicine, Justus Liebig University, Giessen, Germany; 9 Department of Pediatrics, Asklepios Medical School, Hamburg, Germany; 10 Clementine Kinderhospital Frankfurt, Frankfurt, Germany; 11 Division of Pediatric Nephrology, Hepatology and Transplantation, University Medical Center Hamburg-Eppendorf, Hamburg, Germany; 12 Department of Pediatrics I, University Children’s Hospital, Heidelberg, Germany; 13 Department of Pediatrics II, University Children’s Hospital, Philipps-University Marburg, Marburg, Germany

**Keywords:** Guideline, Steroid-sensitive nephrotic syndrome, Frequently relapsing nephrotic syndrome, Steroid-dependent nephrotic syndrome, Treatment

## Abstract

Idiopathic nephrotic syndrome is the most frequent glomerular disease in children in most parts of the world. Children with steroid-sensitive nephrotic syndrome (SSNS) generally have a good prognosis regarding the maintenance of normal kidney function even in the case of frequent relapses. The course of SSNS is often complicated by a high rate of relapses and the associated side effects of repeated glucocorticoid (steroid) therapy. The following recommendations for the treatment of SSNS are based on the comprehensive consideration of published evidence by a working group of the German Society for Pediatric Nephrology (GPN) based on the systematic Cochrane reviews on SSNS and the guidelines of the KDIGO working group (Kidney Disease - Improving Global Outcomes).

**Supplementary Information:**

The online version contains supplementary material available at 10.1007/s00467-021-05135-3.

## Introduction

With an incidence of 1.8 cases per 100,000 children, idiopathic nephrotic syndrome (iNS) is the most common glomerular disease during childhood in Germany [1]. Approximately 90% of all cases are steroid-sensitive with an initial episode successfully treated with a standardized treatment protocol of glucocorticoids (steroids) [2]. However, about 80% of these patients experience further relapses [3, 4]. Of these, 50% relapse frequently or are characterized as steroid-dependent [5]. While any relapse can be treated with steroids [6], children may be vulnerable to the side effects of a high cumulative dose of steroids [7]. Young patients are, for instance, at risk for developing obesity [8], growth impairment [9], behavioral alterations and attention problems [10], as well as reduced quality of life and family stress [11]. To minimize steroid toxicity in patients with steroid-dependent nephrotic syndrome (SDNS) and frequently relapsing nephrotic syndrome (FRNS), a number of immunosuppressive agents other than glucocorticoids are recommended as maintenance therapeutic agents. Among these are cyclosporine, tacrolimus, mycophenolate mofetil (MMF), cyclophosphamide, levamisole, and rituximab. Impaired kidney function is not typically expected and, if so, usually results from nephrotoxic side effects of calcineurin inhibitors. The aim of this guideline is to provide evidence-based recommendations on the diagnosis and differential diagnosis of iNS and on the therapy of steroid-sensitive nephrotic syndrome (SSNS) in childhood. The recommendations will not always be identical to the current recommendations of Kidney Disease: Improving Global Outcomes (KDGIO). Deviations from the KDIGO guidelines will be discussed accordingly.

## Methods

This guideline is based on the definitions and recommendations of the KDIGO guidelines [6] and the Cochrane Collaboration [7, 12, 13]. These international guidelines (KDIGO) and systematic reviews of the existing evidence (Cochrane Collaboration) served as a basis for the development of the recommendations. All published randomized controlled trials (RCTs) on immunosuppressive therapy of the iNS were considered and reevaluated. The evidence level and strength of the recommendations were graded according to the Oxford Centre for Evidence-Based Medicine classification [14] (Supplement Tables 1 and 2).

The GPN commissioned a consensus group to revise the guideline. On August 9, 2019, the coordinators announced the revision of the guideline. A first version of the revision was sent to the consensus group for review on October 1, 2019. The contributions of the different authors were collected in a written circulation procedure and incorporated into a draft. This second draft was extensively discussed in person on November 28, 2019.

The revised third draft of the guideline was again sent to the consensus group for review on March 6, 2020, and discussed in an online conference of the consensus group on May 7, 2020. The revised fourth draft of the guideline was sent to the consensus group for final approval on June 5, 2020. A final consensus was reached on June 19, 2020.

## Definition and delimitation

Childhood nephrotic syndrome (NS) is defined as the occurrence of heavy proteinuria (≥ 40 mg/m^2^ body surface area (BSA)/h or ≥ 1 g/m^2^ BSA/day) combined with hypalbuminemia (< 25 g/L) in serum [15]. Edema is the clinically leading symptom of the disease, but is not obligatory. Secondary hyperlipidemia with an increase in total and low-density lipoprotein (LDL) cholesterol, in severe cases in triglycerides, is usually present. Distinction is made between primary (idiopathic (iNS), genetic) and secondary forms of NS. Secondary and genetic forms are not the subject of this guideline. Please see Table 1 for clinical definitions of NS.

**Table 1:** Clinical definitions of childhood nephrotic syndrome

Classification	Definition
Remission (response)	Proteinuria < 4 mg/m^2^ BSA/h or dipstick (Albustix®) in morning urine negative or trace positive on 3 consecutive days or urinary protein/creatinine ratio < 0.2 g/g
Partial remission	Reduction of proteinuria by ≥ 50%, urinary protein/creatinine ratio < 2 g/g and > 0.2 g/g
Recurrence (relapse)	Recurrence of proteinuria above 40 mg/m^2^ BSA/h (above 1 g/m^2^ BSA/d) or Albustix® dipstick in morning urine ≥ 100 mg/dL (++) on 3 consecutive days or urinary protein/creatinine ratio > 2 g/g
Primary steroid-sensitive NS (initial responder)	Remission induced by a treatment with prednisone 60 mg/m^2^ BSA/d within 4 weeks
Primary steroid-resistant NS (initial non-responder)	No remission after treatment with prednisone 60 mg/m^2^ BSA/d for 4 weeks
Secondary steroid-resistant NS	Primary steroid-sensitive NS, but no response to standard relapse therapy with prednisone (60 mg/m^2^ BSA/d) for a maximum of 4 weeks in later relapses
Infrequently relapsing nephrotic syndrome	Steroid-sensitive nephrotic syndrome with 1 relapse within 6 months after end of therapy or up to 3 relapses within 12 months after end of therapy
Frequent relapses (frequently relapsing nephrotic syndrome, FRNS)	Steroid-sensitive NS with ≥ 2 relapses within the first 6 months after end of therapy or ≥ 4 relapses within 12 months after end of therapy
Steroid-dependent nephrotic syndrome (SDNS)	At least two consecutive relapses under standard relapse therapy with prednisone or within 2 weeks after end of therapy

## Diagnostics

The clinical examination documents in particular the localization and severity of edema, body weight over the course of the disease, arterial blood pressure, and possible abnormalities with respect to a potential syndromic disease or systemic illness.

Laboratory diagnostics are used for the detection of NS with heavy proteinuria, selective proteinuria (urinary albumin content > 80%), and decrease of serum albumin (< 25 g/L). It is important to exclude other causes of proteinuria, especially secondary forms of NS (Table 2). Gross hematuria, arterial hypertension, impaired kidney function, or skin lesions are findings that may be indicative of a secondary form of nephrotic syndrome. The suspicion of a secondary form should lead to a consultation or a transfer to a specialized center.

**Table 2:** Possible causes of secondary nephrotic syndrome according to Benz et al. [16]

Immunological systemic diseases	Systemic lupus erythematosus (SLE), IgA vasculitis with nephritis, IgA nephropathy, granulomatosis with polyangiitis, panarteriitis nodosa, Goodpasture's syndrome, rheumatic fever, sarcoidosis, and others
Infections	Chronic bacteremia (e.g., endocarditis lenta, foreign body infections), hepatitis B and C, infections with cytomegalovirus (CMV) and Epstein-Barr virus (EBV), human immunodeficiency virus (HIV), malaria, schistosomiasis
Tumors	Leukemia, non-Hodgkin’s lymphomas
Hemodynamic	Renal vein thrombosis, congestive cardiomyopathy, sickle cell anemia
Drugs and toxins	Nonsteroidal anti-inflammatory drugs, D-penicillamine, gold, mercury

Urine: Urine status using test strips (dipstick) and microscopy, urine protein/creatinine ratio (uProt/uCrea: concentration of protein and creatinine in urine in g/g) or urine collection for quantitative measurement of protein excretion.

Blood: Complete blood count (CBC), differential blood count; serum: electrolytes, blood urea, creatinine, cystatin C, protein, albumin, serum electrophoresis, liver enzymes, triglycerides, cholesterol; venous blood gas analysis including ionized Ca^2+^.

Immunological parameters: Immunoglobulins A and G, complement proteins C3 and C4.

Coagulation tests: International normalized ratio (INR), partial thromboplastin time (PTT), fibrinogen, antithrombin III.

Indications for thrombophilia screening are persistent hypalbuminemia, thromboembolic complications (current or past), and a positive family history of venous and arterial vessel occlusion in first-degree relatives.

Additional diagnostics that may be required: Thyroid-stimulating hormone (TSH), free thyroxine (fT4), anti-streptolysin titer, antiDNase B, antineutrophil cytoplasmic antibodies (pANCA, cANCA), antinuclear antibodies (ANA), anti-double-stranded DNA antibodies (dsDNA), in suspected membranous glomerulopathy: hepatitis serology to exclude acute or chronic hepatitis B or C, antibody diagnostics (e.g., phospholipase A2 receptor antibody (PLA2R-AK); glomerular basement membrane antibody (GBM-AK)).

Ultrasonography: Detection of normal or enlarged kidneys with normal or elevated echogenicity, detection of ascites and pleural effusions. Exclusion of renal vein thrombosis (difference in size and Doppler ultrasound examination).

X-ray: Chest X-ray only in case of pulmonary symptoms and suspected lymphoma (very rarely associated with secondary NS).

Kidney biopsy: Not initially indicated for typical age of NS manifestation and characteristic course with response to steroids. Kidney biopsy may be indicated in patients aged > 10 years, in case of steroid resistance, nephritic syndrome, or suspected systemic disease.

Differential diagnosis: Exclusion of other diseases, e.g., congestive cardiomyopathy, liver cirrhosis, amyloidosis, protein loss enteropathy, other causes of secondary NS [17]. Please see Table 2.

For guidance on genetic testing in the presence of a steroid-resistant course, please see guidelines on “steroid-resistant nephrotic syndrome in childhood” [18].

## Therapy of the initial manifestation of an iNS

We recommend treatment of the initial manifestation of an idiopathic NS (iNS) with prednisone in the dosage of 60 mg/m^2^ body surface area (BSA)/d orally (administered in a single daily dose, maximum 80 mg/d) for 6 weeks, followed by the alternating administration of prednisone at a dose of 40 mg/m^2^ BSA/d orally (in a single daily dose, maximum 60 mg/d) for another 6 weeks (level of evidence 1B for therapy duration of 12 weeks).

### Comment

Drug treatment of iNS is imperative, as increasing edema (e.g., ascites, pleural effusion, pericardial effusion, pulmonary edema) can become life-threatening and, in particular, the occurrence of acute kidney failure and bacterial infections are associated with high mortality.

The standard therapy with steroids, developed empirically by the ISKDC in the 1970s [19] and later modified by the *Arbeitsgemeinschaft für Pädiatrische Nephrologie* — Working Group on Pediatric Nephrology (APN) [20], originally envisaged a treatment duration of 8 weeks. Randomized controlled clinical trials (RCTs) in children have confirmed the efficacy of this therapy and further modifications have been reviewed. Since 2000, all published RCTs have been systematically evaluated by the Cochrane Group in a meta-analysis [6]. In the last update of this meta-analysis [13], the evidence of these studies was evaluated as follows:

- A treatment duration of 12 weeks compared to 8 weeks leads to a 20% reduction in the risk of relapse (within 12–24 months) (8 studies with a total of 741 children) and a 32% reduction in the risk of frequent relapses (5 studies, 582 children) (evidence level 1B).
- An extended treatment duration of 5–6 months leads to a 38% reduction in the risk of relapse (7 studies, 763 children), but not to a reduction in the risk of frequent relapses (5 studies, 591 children) within 12–24 months.

Discussing the results, the authors of the Cochrane review point out the heterogeneity of the latter studies, suggesting that extended treatment duration (of more than 12 weeks) is unlikely to be associated with a significantly reduced risk of relapse. There is evidence from a number of more recent RCTs speaking against treatment extension beyond 8 [4, 21] or 12 weeks [3, 22], led by a Dutch study showing that extending initial prednisolone treatment from 3 to 6 months without increasing cumulative dose did not benefit clinical outcome in children with nephrotic syndrome [3]. In addition, a study by the Japanese Society of Kidney Disease in Children [21] found no difference in the risk of frequent relapses with 2 or 6 months of therapy (RCT, 255 children). Another study in India [22] also found that extending standard therapy from 12 weeks for a further 3 months did not improve the outcome (RCT, 181 patients).

These results are also confirmed by the double blind, placebo-controlled PREDNOS study in the UK [4, 23]. There were no differences between an 8-week versus a 16-week initial treatment with prednisolone with regard to the occurrence of the first relapse and the development of a course with frequent relapses. In summary, there is little rationale for a duration of prednisone therapy longer than 12 weeks in the treatment of the initial manifestation of a SSNS, which thus appears unnecessary according to the principle of primum nihil nocere.

The KDIGO recommendations of 2012 further differentiate the level of evidence with respect to treatment in the first and second half of treatment:

- Since the duration of remission was not significantly different in initial therapy with a single daily dose of prednisone compared to splitting the daily dose into multiple doses, the KDIGO authors recommend the administration of prednisone as a single dose for 6 weeks (level of evidence 1 B). The division of the daily dose into 3 single doses established in Germany should no longer be propagated for reasons of adherence. Daily prednisone administration is recommended for 4–6 weeks (evidence level 1C). In Germany, administration over 6 weeks is established.

The recommended dosage during daily administration is 60 mg/m^2^ BSA/d; during alternate administration it is 40 mg/m^2^BSA/d (evidence level 1D). KDIGO recommends a limitation of the maximum daily dose to 60 mg (daily administration) or 40 mg (alternating administration). The maximum doses established in Germany are higher (80 mg for daily and 60 mg for alternate administration), and it is not known whether the associated higher cumulative dose has a significant effect. Current efforts are aiming at reducing the cumulative steroid dose. However, since the evidence level is low (D), we see no reason to change the maximum daily doses established in Germany, which are also still used in current RCTs. The currently available evidence from clinical trials does not suggest a change in the recommended regimen for treatment of the initial manifestation of the iNS. Therapy with lower prednisone doses (40 mg/m^2^ BSA/d for 6 weeks, followed by alternating doses for another 6 weeks) [23] was associated with an increased relapse rate in boys in a single study, but not in girls (n = 26) compared to therapy with 60 mg/m^2^ BSA/d [24]. However, these data from a small subpopulation in a single study do not appear to be sufficient to justify lower steroid dosage in initial therapy. Drug intensification of the initial therapy, i.e., the administration of high-dose oral methylprednisolone [24] or the additional administration of azithromycin (antibiotic with immunomodulatory and anti-inflammatory effects) [25] or cyclosporine (150 mg/m^2^/d for 8 weeks) [26] did not show any advantage in terms of a clinically relevant prolonged duration of remission.

There is no need to taper the steroids at the end of the recommended alternating therapy. However, supraphysiological steroid therapy, as performed in the treatment of iNS, carries the risk of suppression of the hypothalamic-pituitary-adrenal axis with transient central adrenal insufficiency after termination of prednisone therapy [27, 28]. There is no data on the duration and frequency of complications of transient central adrenal insufficiency in childhood NS. Possible clinical signs of secondary adrenal insufficiency may be anorexia, nausea, vomiting, abdominal pain, weakness, fatigue, myalgia, arthralgia, weight loss, hypotension, somnolence, and depression. Signs of acute adrenal crisis may include vomiting, diarrhea, fever, acute dehydration, hypotension, hypoglycemia, shock, and coma. Especially in the case of infections and fever, signs of central adrenal insufficiency should be considered in the first 8 (-12) weeks after termination of steroid therapy and, if necessary, substitution should be considered with hydrocortisone in “stress dose” of 30 mg/m^2^ BSA per day in 3 doses. If there is evidence of an acute adrenal crisis, high-dose intravenous hydrocortisone (under regular electrolyte control) should be administered [29].

## Therapy of relapses

### Infrequent relapses

We suggest that children and adolescents with infrequent steroid-sensitive NS (SSNS) be treated with oral prednisone at the dose of 60 mg/m^2^ BSA/d (in a single daily dose, maximum 80 mg/d) until the urine is protein-free for 3 consecutive days (level of evidence 2D). Prednisone therapy is then to be continued for 4 weeks at 40 mg/m^2^ BSA/48 h (in a single alternating dose, maximum 60 mg) (level of evidence 2C).

#### Comment

In a study in children with SSNS, it was shown that after the onset of remission, alternate therapy (prednisone administration every 2nd day) over 4 weeks was as effective (in terms of the prevention of relapses in the following 9 months) as daily prednisone administration over 8 weeks [5]. From this study, the currently recommended therapy for infrequent relapses was empirically derived; other RCTs are missing [30]. Despite this relatively sparse evidence [5], the recommendation may be considered acceptable, as the treatment is short-term and steroid toxicity does not usually occur at this dose. However, if frequent relapses (FRNS) or steroid dependence (SDNS) develop, alternative steroid-sparing treatment measures should be considered (see below). We follow the KDIGO recommendations, but with modification of the prednisone dose (higher maximum daily dose) for the same reasons as with the initial therapy of the iNS.

In 23% of relapses of children with frequently relapsing NS and in 10% of those with steroid dependence, spontaneous proteinuria remissions without steroid therapy are observed. Therefore, in the case of a relapse, one can try to delay steroid therapy for about 7–10 days depending on the clinical situation, especially in the case of relapses associated with infections. In cases of severe proteinuria (Albustix® ++++) or increasing edema and a significant increase in weight (> 1 kg or > 5% above the initial weight), treatment with prednisone should be finally started.

Whether the duration of alternating prednisone administration can be reduced from 4 to 2 weeks without reducing efficacy is currently being investigated prospectively (RESTERN study, EudraCT 2016- 002430-76; [31]).

### Frequent relapses of steroid-sensitive nephrotic syndrome (FRNS) and steroid-dependent nephrotic syndrome (SDNS)

#### Therapy with glucocorticoids

We recommend that children and adolescents with frequent relapses of steroid-sensitive NS (FRNS) or with steroid dependence (SDNS) be treated with prednisone in the dosage of 60 mg/m^2^ BSA/d (in a single daily dose, maximum 80 mg/d) until the urine is protein-free for 3 consecutive days. Subsequently, prednisone therapy is to be continued for 4 weeks at 40 mg/m^2^ BSA/48 h (in a single every other day dose, maximum 60 mg/d) (level of evidence 2C).

#### Comment

The practical implementation of therapy is not different from the therapy of infrequent relapses (see above); however, with increasing duration of therapy and diagnosis of FRNS or SDNS as well as increasing clinical signs and/or the risk of steroid toxicity, the indication for steroid-sparing therapy (see below) arises for these patients.

Since the APN was able to show in 1981 that alternating therapy (1 dose of prednisone every 2nd day) is significantly more effective in maintaining iNS remission than intermittent administration (3 doses per week followed by a 4-day break), this therapy has become established in German-speaking countries as well as internationally [20].

In addition, in some countries there are treatment recommendations for maintaining remission with low-dose steroids as well as with a short-term increase in the steroid dose in the event of a viral infection.

The treatment with prednisone in the “lowest dose required” to maintain remission, which is common in many countries, has been investigated in observational studies on relatively small numbers of patients; RCTs are not available. At doses of 0.48 mg/kg body weight (BW) on alternating days or 0.25 mg/kg BW daily, the occurrence of relapse was significantly reduced compared to historical controls [32, 33]. The British Association of Paediatric Nephrology and the Indian Pediatric Nephrology Group recommend long-term low-dose administration of prednisone in FRNS to prevent relapse. The KDIGO guidelines also suggest (level of evidence 2D) to use the lowest possible steroid dose to maintain remission and, if an alternating dosing regimen is not successful, to switch to daily administration of the same dose [6]. Since the evidence is low (no RCTs) and the empirically-based recommendations are not proven, this approach can only be considered in individual cases.

Since clinical observation shows that intercurrent infections, especially respiratory infections, can trigger relapses of NS, a short-term increase in steroid dose is practiced during such infections. A total of 3 RCTs have demonstrated a significant benefit of this measure, but only for patients who were already receiving alternating standard relapse therapy or low-dose long-term therapy with prednisone, sometimes also in combination with levamisole, at the onset of infection [34–36]. The KDIGO guidelines suggest (evidence level 2C) that patients on alternate steroid therapy should switch to daily administration for the duration of intercurrent respiratory tract infection. The evidence base, however, is incomplete. Since long-term low-dose therapy with steroids is hardly practiced in Germany and is not recommended by us, a possible indication may arise in individual cases only. We therefore do not make a general recommendation for an increase in the steroid dose in respiratory tract infections.

The short-term administration of steroids for the prophylaxis of a relapse in case of upper respiratory tract infections cannot be generally recommended even in children who are under long-term steroid therapy, but may be considered in individual cases. Logistical difficulties in identifying the indication must also be considered. Although a prospective, double-blind cross-over study from Sri Lanka showed that patients with upper respiratory tract infection benefited from a 5-day administration of 0.5 mg/kg BW prednisolone per day in terms of a reduction in relapse probability [35], interpretation of the results is complicated due to a high dropout rate. In addition, there is no analysis of the cumulative steroid dose between treatment groups, and in the placebo group, about 75% of upper respiratory tract infections did not lead to relapse. In the future, the results of a prospective randomized British study may contribute to the value of short-term steroid administration in upper airway infections [37].

## Steroid-sparing immunosuppressive agents for frequent relapses and/or steroid dependence

The principle of primum nihil nocere applies to all steroid-sparing drugs; they should be reserved for patients who have developed steroid-associated side effects. In the case of a steroid-dependent course (SDNS), an earlier decision for a steroid-sparing long-term therapy may have to be made, since steroid-associated side effects can develop more rapidly than in the case of frequent relapses (FRNS). Several substances are available for steroid-free immunosuppression (Table 3).

**Table 3:** Steroid-sparing immunosuppressive agents for frequent relapses and/or steroid dependence

	Indication in the scientific information	Recommended dose	Side effects	Monitoring	Therapy duration	Advantage/indication
Cyclosporine	Steroid-dependent and steroid-resistant nephrotic syndrome resulting from primary glomerular diseases such as minimal change nephropathy, focal segmental glomerulosclerosis or membranous glomerulonephritis	150 mg/m^2^ divided into two oral doses, in the further course adjustments after blood trough levels might be necessary	Kidney dysfunction, tremor, hypertrichosis, hypertension, diarrhea, anorexia, nausea and vomiting, gingival hyperplasia	Blood trough level (80–120 ng/mL initial, later lower, 50–80 ng/mL) Serum Creatinine	1–4 years	Good effectiveness in long-term therapy
Tacrolimus	Off-label	0.1–0.15 mg/kg divided into two oral doses, in the further course adjustments after blood trough levels might be necessary	Kidney dysfunction, tremor, hypertension, diarrhea, anorexia, nausea, vomiting, diabetes mellitus	Blood trough level 3–5 (-8) ng/mL Serum Creatinine	1–4 years	Good effectiveness in long-term therapy, fewer cosmetic side effects (gingival hyperplasia, hypertrichosis)
Mycophenolic acid	Off-label	1200 mg/m^2^ divided into two oral doses, in the course adjustments according to MPA-AUC might be necessary	Diarrhea and vomiting, leukocytopenia, sepsis, increased infection rate. Contraindicated in pregnancy	Blood count controls, plasma predose concentration, if necessary determination of total exposure (AUC kinetics) for individual dose finding	1–4 years	Good effectiveness with adequate exposure
Cyclophosphamide	Threatening “autoimmune diseases,” severe, progressive forms of lupus nephritis and granulomatosis with polyangiitis (GPA)	2–3 mg/kg per day in one oral dose for 8–12 weeks	Myelosuppression especially leukocytopenia, hemorrhagic cystitis	Blood count checks initially weekly	8–12 weeks	Short therapy duration,potentially permanent remission
Levamisole	Off-label	2–2.5 mg/kg every second day as a single oral dose (max. 150 mg)	Leukocytopenia, allergic reactions, gastrointestinal disorders, skin necrosis, ANCA-positive vasculitis	First weekly blood count, then at 4–12 weekly intervals	1.5–2 years	Efficacy especially for frequent relapses, less for steroid dependence
Rituximab	Off-label	375 mg/m^2^ intravenously as a single dose	Potentially fatal infections, neutropenia, drop in IgG and IgM, skin reactions, cytokine release syndrome, progressive multifocal leukoencephalopathy	Blood count controls, control of immunoglobulins G and M, prophylaxis of Pneumocystis jirovecii infection	If necessary, repeat the application during the course of the treatment (e.g., after B-cell monitoring)	Good efficacy, side effect profile in this indication still insufficiently documented. Only indicated if the usual therapy is not sufficiently effective

### Calcineurin inhibitors

#### Cyclosporine

We recommend the administration of cyclosporine in the treatment of frequently relapsing and steroid-dependent NS (level of evidence 1B) [38–42].

#### Comment

In cases of frequent relapses or steroid-dependent nephrotic syndrome, treatment with cyclosporine at an initial dose of 150 mg/m^2^ BSA/day divided into two oral doses after remission induction with prednisone is recommended. Initially, blood trough levels of about 80–120 ng/mL are typically targeted. In long-term therapy, the dose should be slowly reduced to the lowest effective dose under control of blood trough levels [41].

### Tacrolimus

We recommend the administration of tacrolimus in the treatment of frequently relapsing NS or steroid-dependent NS (level of evidence 2B).

#### Comment

Tacrolimus has not been investigated in randomized prospective studies in steroid-sensitive NS so far, but only in non-controlled observational studies [43, 44]. The recommended dose of tacrolimus in steroid-sensitive NS is 0.1–0.15 mg/kg BW divided into two oral daily doses. In long-term therapy a trough level of 3–5 ng/mL is aimed for; in exceptional cases, up to 8 ng/mL.

### Mycophenolate mofetil (MMF)/mycophenolic acid (MPA)

We recommend the administration of MMF in the treatment of frequently relapsing NS and steroid-dependent NS (level of evidence 1B) [40, 42, 45–56].

#### Comment

We recommend the following MMF dosage for steroid-sparing monotherapy and concomitant therapeutic drug monitoring (level of evidence 2B):

- 1200 mg/m^2^ BSA per day divided into two oral daily doses
- Start of therapy, e.g., already under alternating steroid therapy
- Target range: MPA-AUC_0-12_ > 50 mg × h/L

Therapeutic drug monitoring can be performed in patients with MMF monotherapy using a strategy adapted to the outpatient care situation with three serial blood samples taken over 2 h (abbreviated pharmacokinetic profile with plasma MPA determinations at times 0 min (before administration, C_0_), 60 min (C_1_), 120 min (C_2_) after administration), which allows a good estimation of MPA-AUC_0-12_ [57]:

$$ \mathrm{eMPA}-{\mathrm{AUC}}_{0-12}=8.70+4.63\ast {\mathrm{C}}_0+1.90\ast {\mathrm{C}}_1+1.52\ast \kern0.5em {\mathrm{C}}_2 $$

Of note, MMF is teratogenic; for women of childbearing age and men of reproductive age, respectively, effective contraception is recommended by the manufacturer prior to initiation of treatment up to 6 weeks after cessation of treatment for women and up to 90 days after cessation of treatment for men. Registry studies have shown no effect of paternal use of MMF on teratogenicity compared with the normal population [54, 55, 58]. The use of enteric-coated mycophenolate sodium (EC-MPS) in children and especially in childhood nephrotic syndrome is very rare, which is due to the lack of a liquid formulation and the lack of studies on EC-MPS and nephrotic syndrome.

### Cytostatic drugs (cyclophosphamide)

In Germany, the use of cyclophosphamide has been abandoned due to the associated side effects in favor of treatment alternatives.

We suggest considering the use of cyclophosphamide in individual cases of frequent relapses or steroid-dependence if other alternatives with fewer side effects have been used unsuccessfully.

#### Comment

Cyclophosphamide should be given at a dose of 2–3 mg/kg/day in a single oral dose for 8 to 12 weeks; the maximum cumulative dose of 168 mg/kg should not be exceeded (level of evidence 2C). The advantage of a possible permanent remission after administration of cyclophosphamide is counterbalanced by a higher risk of considerable side effects [38, 59–65]. The major concern is the dose-dependent impairment of spermatogenesis up to azoospermia in male patients [65]. Although a cumulative dose of cyclophosphamide of 200 mg/kg is generally accepted as a threshold for a high risk of gonadal toxicity, a precise estimate of the long-term risk does not appear possible, since an individual risk can also be assumed below this threshold and there are also documented cases of azoospermia at lower doses [65].

### Levamisole

We suggest considering the use of levamisole in the treatment of frequently relapsing NS, especially when there is no steroid dependence (level of evidence 1B). The dose should be 2–2.5 mg/kg BW, administered on every second day [66–73].

### Rituximab

We suggest the administration of rituximab only in patients with complicated courses of a steroid-sensitive iNS (especially relapses under immunosuppressive maintenance therapy with calcineurin inhibitors and/or MMF) or severe side effects under these drugs (level of evidence 1B) [39, 60, 74–85]. The use, monitoring, and follow-up of rituximab therapy should be reserved for specialized pediatric nephrology centers.

The efficacy of rituximab in SSNS (but not in steroid-resistant NS) has been demonstrated by various registry data and prospective studies [80]. Good efficacy was documented in a randomized placebo-controlled trial (level of evidence 1B) from Japan [85]. A prospective, randomized parallel study from India [74] showed that rituximab versus tacrolimus resulted in fewer relapses (relapse-free survival at 1 year 90% versus 63.3%) and steroid-sparing in patients with SDNS who had not previously received other immunosuppressive alternative medications. However, the results must be interpreted in light of the following limitations: different durations of concomitant steroid therapy in the treatment arms (4 weeks under rituximab, 6 months under tacrolimus), potential ethnic and geographic influencing factors, relatively short observation period of 12 months. Patients received two doses of rituximab (375 mg/m^2^ BSA each, maximum 500 mg) 1 week apart. At the end of observation after 12 months, 93.2% of the patients already had normal B cells again. Moreover, the number of B cells was higher in patients with relapses than in those without relapse during the observation period (see below). Thus, it remains unclear whether the superiority with respect to relapse prophylaxis over tacrolimus is maintained beyond the first year of observation or necessitates further rituximab administration. It is thus also unclear whether repeated administrations would not shift the comparably good tolerability to the disadvantage of rituximab. Before rituximab can be considered as first-line therapy in SDNS, further prospective studies must demonstrate its efficacy and tolerability over a longer observation period.

Many questions about treatment with rituximab also remain unanswered. Regarding the initial dose and repetition of treatment cycles, a French publication recommended monitoring of B cells and (in some cases multiple) repetition of rituximab administrations to maintain B cell depletion [83]. However, it was shown that an initial course of 1–2 infusions was comparable to a course of 3–4 initial administrations in terms of achieving long-term remission [79]. Other studies support that a single infusion at baseline can maintain remission; comparative studies of different dosing intervals are not yet available [82].

The dose is 375 mg/m^2^ BSA administered intravenously after steroid-induced remission is achieved. Rituximab should not be given during an acute infection. Florid hepatitis B infection is an absolute contraindication and must be ruled out before rituximab therapy.

Currently, it is unclear whether repeated administration of rituximab (1–4 administrations in the literature) improves long-term outcome. The primary goal should be cessation of steroid therapy and concomitant medication, but in some studies, immunosuppression, e.g., with MMF [86], has been continued. In case of recurrence and especially recurrence of steroid dependence, repeated rituximab infusions may be indicated later in the course.

Administration of chemoprophylaxis against *Pneumocystis jirovecii* with cotrimoxazole is recommended until normalization of CD20+ lymphocytes.

Efforts should be made to minimize the number of rituximab administrations. There are currently insufficient data for automatic repeated doses to maintain persistent B-cell depletion (an increase in CD20+ lymphocytes usually occurs after 6–9 months, but does not automatically lead to relapses). Immunological monitoring is recommended (differential blood count, number of CD19-positive B lymphocytes, immunoglobulin G and M concentrations in plasma every 3 months until normalization). In case of hypogammaglobulinemia (often detectable in NS even without rituximab therapy) and recurrent or severe infections, IgG substitution may be necessary.

## Supportive therapy and complications

In general, sufficient sun protection is recommended for patients with long-term immunosuppressive therapy [87]**.** Suitable measures are, e.g.:

- Refrain from sunbathing and solarium visits
- As little exposure to UV radiation as possible at times of very high UV exposure (11 am to 15 pm)
- Wear appropriate clothing (cover as much skin area as possible: “shirt, hat and trousers”)
- Use of sun protection creams with high to very high sun protection factor (SPF 30 to 50)

Edematous patients are overfilled with sodium and water, so that, especially in the case of pronounced edema, a reduction of saline and fluid are basic components of the therapy in order to prevent further edema formation. This requires exact fluid balancing. Since only the intravasal/circulating blood volume can be reduced primarily, therapy with diuretics must be carried out carefully to avoid further intravascular volume depletion. Furosemide (dosing range 1–2–5 mg/kg/d) is administered orally or intravenously; in more severe cases (oligoanuria, therapy-resistant edema, pronounced ascites, anasarca, or nephrotic crisis) following an albumin infusion, a dose of 1 g/kg albumin 20% intravenously over 1–2 h), followed by 0.5–1 mg/kg BW i.v. furosemide. Close monitoring is indicated.

Immobilization should be avoided at all costs; if bed rest is necessary and/or other thrombophilic factors (see below) are present, thrombosis prophylaxis is advised. Central venous catheters should be avoided, if possible, due to the augmented risk of thrombosis.

In the presence of arterial hypertension, the administration of an angiotensin-converting enzyme (ACE) inhibitor or an angiotensin (AT)-II receptor blocker is recommended. However, caution is advised in case of intravascular volume deficiency.

## Complications

NS can be accompanied by serious complications, especially in steroid-resistant forms with prolonged hypoalbuminemia. A distinction is made between acute complications in the phase of nephrotic proteinuria (e.g., thromboembolism, infections, pre-renal acute kidney failure, pulmonary edema) and complications that are caused by the long-term course or its therapy (e.g., osteoporosis, dwarfism, and increased cardiovascular risk) [16]. Approximately one-third of all patients who develop SSNS in childhood continue to have active disease in early adulthood, and patients with SDNS or FRNS are particularly at risk for persistent disease [88].

### Thromboembolism

The risk of thromboembolism is significantly increased in children with NS. The incidence is reported to be as high as 2–5% [89], and even higher in complicated NS [90].

An underlying congenital thrombophilia significantly increases the risk. Therefore, targeted thrombophilia screening should be performed after the occurrence of thromboembolic complications and especially in the case of a positive family history of venous and arterial vessel occlusion in first-degree relatives [91]. Typical locations of thrombosis are the sinus veins, pulmonary veins and right atrium, deep leg veins, and kidney veins. However, arterial thrombosis can also occur [92].

The following coagulation tests can be used to detect hereditary and acquired coagulation disorders: protein C, protein S, antithrombin III, APC (activated protein C) ratio, lipoprotein (a), prothrombin mutation G20210A, cardiolipin antibodies IgM/IgG, beta 2 glycoprotein antibodies IgM/IgG and lupus anticoagulants. It is advisable to perform or repeat this diagnostic procedure in a period of remission. Since there are no studies or clear criteria for the prophylactic application of anticoagulants in addition to early mobilization of the patient, prophylaxis with low molecular weight (LMW) heparin can be discussed individually. Dispositional (diagnosed thrombophilia, known personal or family history of thrombosis, congenital nephrotic syndrome, cardiovascular comorbidity and sepsis or severe infection) and expositional (hyperviscosity/hypovolemia, thrombocytosis, prolonged acute phase (increase in plasma concentration of F I, V, VIII), hyperlipidemia, loss of antithrombin III or a central vein catheter) risk factors can help to estimate the individual risk profile for a thromboembolic complication and thus for drug prophylaxis.

In summary, a general recommendation for thrombosis prophylaxis cannot be given; an individual decision under evaluation of the current risk factors is necessary. A level-controlled anticoagulation with low-molecular-weight heparin (prophylactic target level: anti-Xa activity 0.2–0.4 U/L) (cave: extension of half-life in the case of impaired kidney function and delayed renal clearance; therefore, adjustment of the dose to the estimated glomerular filtration rate (eGFR) is necessary) is recommended rather than therapy with acetylsalicylic acid or oral anticoagulation with vitamin K antagonists.

### Infections

Infections due to secondary antibody deficiency, reduced cellular and humoral immunity, and possibly immunosuppressive therapy are rare but often serious. Prior to the introduction of steroid therapy, infections, especially by *Streptococcus pneumoniae*, were the main causes of death in children with NS.

Favored by accumulation of serous fluid, there is an increased risk of dermal phlegmons, empyema, and ascites peritonitis, caused mainly by *Staphylococcus aureus* and *Streptococcus pneumonia* [93]. Sepsis, meningitis, or pneumonia also occur more frequently, so that early antibacterial therapy is indicated in suspected cases. There is no evidence for a general antibacterial prophylaxis. Children with NS should be vaccinated sequentially with the pneumococcal conjugate vaccine as well as with the 23-valent pneumococcal polysaccharide vaccine and annually against influenza [94].

Live vaccinations under immunosuppressive therapy are usually contraindicated. However, varicella especially can cause life-threatening diseases in patients under immunosuppressive therapy. If a chronic or chronic-relapsing disease starts early in life, the recommended vaccination plan may not be implemented. In the course of the disease, the question arises whether the stage of disease or the extent of immunosuppressive therapy could allow a vaccination after careful consideration and whether such a vaccination could lead to seroconversion [95]. Vaccinations in patients in remission without any immunosuppressive therapy are safe and advised. In all other cases, we refer to the German expert group for vaccination in case of immunodeficiency [96]. A short summary of these recommendations can be found in Supplement Table 3. As these are German guidelines, they are by no means general recommendations and any existing national guidelines have to be respected.

### Pulmonary edema

Therapy-refractory, severe edema is sometimes associated with pulmonary edema, especially in patients with acute kidney failure or oliguria. In this case, albumin infusions must be carried out with extreme caution as they can lead to a redistribution of peripheral edema fluid into the pulmonary circulation.

### Late complications

Long-term use of glucocorticoids or intensified immunosuppressive therapy can lead to growth failure (15%), osteoporosis (13–63%), obesity (5–23%), cataract (6–20%), and arterial hypertension (6–46%) in a significant proportion of patients [8, 97–101].

### Cardiovascular diseases

Patients with frequent relapses or steroid-resistance have an increased risk of developing cardiovascular disease due to the long duration of the disease and side effects of drug therapy. Arterial hypertension, dyslipidemia, long-term therapy with glucocorticoids and calcineurin inhibitors, and the development of chronic kidney disease in (secondary) steroid-resistant forms of NS are associated risk factors.

### Psychosocial aspects and transition to adult medicine

Actually, the full participation of affected children in school and out-of-school life should be aimed for. Regardless of the therapy, in the case of chronic recurrent disease, the quality of life of children with NS and their families is reduced and the psychosocial burden is increased [100, 102]. In the sense of a holistic therapy approach, family training for NS was developed in Germany as a psychological-pedagogical intervention with medical contents. This modular training program provides children with NS and their family members with knowledge relevant to everyday life and information about dealing with the disease (https://www.pabst-publishers.com/fileadmin/user_upload/_modus_9783899678987/modus_9783899679076.pdf).

The transition process, which is completed with the transfer to adult nephrology, should be planned early, ideally including family training at various ages.

As there is an increased risk of developing chronic kidney disease in adulthood even without recurrence after puberty, regular examination by an adult nephrologist is recommended [103].

### Further complications

Further rarely observed complications are hypovolemic shock and intussusception, and in long-lasting NS anemia, hypothyroidism (caused by kidney loss of thyroxine-binding globulin; at normal T3 and T4 concentrations, children are considered normothyroid), and vitamin D deficiency (caused by kidney loss of vitamin D-binding globulin).

## Concluding remarks

The early affiliation of patients with an NS to a practice or clinic with a focus on pediatric nephrology should be considered, since more than half of patients will experience a complicated course, but also to give them the opportunity to participate in therapy studies. For example, the GPN is currently conducting a multicenter study on the initial treatment of iNS to evaluate the efficacy of reduced steroid exposure in the initial treatment of SSNS in children (initial treatment of idiopathic nephrotic syndrome in children with mycophenolate mofetil vs. prednisone: a randomized, controlled, multicenter study, INTENT Study, EudraCT No.: 2014-001991-76) [104].

Specialized institutions with a focus on pediatric nephrology should care for patients with FRNS or SDNS as well as steroid-resistant NS. The risk of potentially serious side effects as well as complications in the course of the disease often requires years of consistent care in a center for pediatric nephrology to avoid long-term organ damage. Reference to local or national patient support groups should be considered.

## Supplementary Information

ESM 1 (PDF 674 kb)

## Data Availability

Not applicable
