# Supplementary material for: Pediatric idiopathic steroid-sensitive nephrotic syndrome: diagnosis and therapy —short version of the updated German best practice guideline (S2e) — AWMF register no. 166-001, 6/2020
Source: Pediatr Nephrol. 2021 Jun 6;36(10):2971–85. doi: 10.1007/s00467-021-05135-3 (PMC8445869; doi:10.1007/s00467-021-05135-3)
Supplement: Supplementary file 1 — (PDF 674 kb) [file 467_2021_5135_MOESM1_ESM.pdf]

Pediatric Idiopathic Steroid-sensitive Nephrotic Syndrome: Diagnosis and Therapy

Short version of the updated German Best Practice Guideline (S2e)

AWMF register no. 166-001, 6/2020

Rasmus Ehren, Marcus R. Benz, Paul T. Brinkkötter, Jörg Dötsch, Wolfgang R. Eberl, Jutta Gellermann, Peter F. Hoyer, Isabelle Jordans, Clemens Kamrath, Markus J. Kemper, Kai Latta, Dominik Müller, Jun Oh, Burkhard Tönshoff, Stefanie Weber, Lutz T. Weber on behalf of the German Society for Pediatric Nephrology

Corresponding author:

Dr. Rasmus Ehren
Pediatric Nephrology, Children's and Adolescents' Hospital, Cologne, Germany
Kerpener Str. 62, 50937 Köln, Germany
Phone: +49 (0)221 - 4784319
Fax: +49 (0)221 – 4785835
Email: rasmus.ehren@uk-koeln.de

Supplementary Information

Supplement table 1: Classification of literature according to the "classes of evidence" (Oxford Centre for Evidence-based Medicine, 2009 [1])

1a	Systematic review (with homogeneity) of randomized controlled trials (RCTs)
1b	Individual RCT (with narrow confidence Interval)
1c	All or none principle
2a	Systematic review (with homogeneity) of cohort studies
2b	Individual cohort study (including low quality RCT; e.g., <80% follow-up)
2c	"Outcomes" Research; Ecological studies
3a	Systematic review (with homogeneity) of case-control studies
3b	Individual Case-Control Study
4	Case-series (and poor quality cohort and case-control studies)
5	Expert opinion without explicit critical appraisal, or based on physiology, bench research or "first principles"

Supplement table 2: The strength of recommendations using the GRADE approach [2, 3]

Grade	Implications		
	Patients	Clinicians	Policy
Level 1 "We recommend"	Most people in your situation would want the recommended course of action and only a small proportion would not.	Most patients should receive the recommended course of action.	The recommendation can be evaluated as a candidate for developing a policy or a performance measure.
Level 2 "We suggest"	The majority of people in your situation would want the recommended course of action, but many would not.	Different choices will be appropriate for different patients. Each patient needs help to arrive at a management decision consistent with her or his values and preferences.	The recommendation is likely to require substantial debate and involvement of stakeholders before policy can be determined.

Supplement table 3: Adapted short summary of the German guideline on vaccination of immunocompromised patients [4] focused on immunosuppressive therapy potentially used in pediatric idiopathic nephrotic syndrome (BSA = body surface area; BW = body weight; MMR = measles, mumps and rubella; HPV = human papilloma virus; MMR-V = measles, mumps, rubella and varicella; PPSV23 = pneumococcal polysaccharide vaccine 23-valent; SmPC = summary of product characteristics)

Drugs	Inactivated vaccination	Live vaccination	Comments
Glucocorticoids (prednisolone equivalent)<br><br>Children<br>*Low level immunosuppression*:<br>Short term therapy (<2 weeks) or low dosage (<0.2 mg/kg BW/day<br>or <10 mg/day)<br><br>*High level immunosuppression:*<br>≥0,2 mg/kg BW/day or<br>≥10 mg/day over ≥2 weeks<br>or intravenous pulse therapy<br><br>Adults<br>*Low level immunosuppression:*<br>Short-term therapy<br>(<2 weeks) or low<br>dosage (<10 mg/day)<br><br>*High level immunosuppression:*<br>≥10 mg/day over ≥2 weeks<br>or<br>intravenous high-dose therapy	Possible at any time.<br>Ideally complete immunization<br>at least 2, better 4 weeks before the start of therapy.	Dose dependent<br><br>*High-dose therapy:*<br>All live vaccines are contraindicated during therapy.<br>Vaccinations must be completed at least 2 better 4 weeks before therapy start.<br><br>*Low-dose therapy:*<br>MMR, MMR-V, varicella vaccination possible during therapy	During short-term therapy (<2 weeks) or at low dosages no relevant effect on safety or effectiveness of a vaccination. For a therapy duration of ≥2 weeks<br>with higher doses or intravenous high-dose therapy,<br>the immunogenicity of vaccinations can be subsequently limited for 2-4 weeks.<br>Significantly limited safety of live vaccines in high-dose glucocorticoid long-term therapy<br>(≥2 weeks).

Cyclosporine<br><br>*Dosing in children and adults:*<br>Low: ≤2,5 mg/kg BW/day<br>High: <2,5 mg/kg BW/day	Possible at any time. Ideally complete immunization at least 2, better 4 weeks before the start of therapy.	<u>Dose dependent</u><br><br>*High-dose therapy:*<br>Contraindicated during therapy, vaccinations at least 4 weeks before and at the earliest 3 months after therapy.<br><br>*Low-dose therapy:*<br>According to expert consensus and in accordance with the pediatric guideline of the European League Against Rheumatism, MMR, MMR-V or varicella vaccinations with Priorix®, PriorixTetra® or Varilrix® can be considered after individual risk-benefit assessment (off-label use due to special warnings in the SmPC).	At low doses (monotherapy) no significant reduction of the response to vaccination. Combination therapy with prednisolone and methotrexate reduces immune response to influenza-vaccination.
Cyclophosphamide	Possible at any time. Ideally complete immunization at least 2, better 4 weeks before the start of therapy.<br><br>During ongoing therapy: Administration in the middle of the treatment interval.	Contraindicated during therapy, vaccinations at least 4 weeks before or at the earliest 3 months after therapy.	

Mycophenolate mofetil<br><br>*Dosing in children:*<br>Low: ≤1200 mg/m² BSA/day<br>High: >1200 mg/m² BSA/day<br><br>*Dosing in adults:*<br>Low: ≤2000 mg/day<br>High: >2000 mg/day	Possible at any time.<br>Ideally complete immunization at least 2, better 4 weeks before the start of therapy.	Dose dependent<br><br>*High-dose therapy:*<br>Contraindicated during therapy, vaccinations at least 4 weeks before and at the earliest 2 months after therapy.<br><br>*Low-dose therapy:*<br>According to expert consensus, MMR-, MMR-V or varicella vaccination with Priorix®, PriorixTetra® or Varilrix® could be considered on an individual risk-benefit assessment (off-label use because of special warnings in SmPC).	Reduced immune response to influenza or HPV vaccination.<br>No significant reduction of the vaccination response at low doses.
Rituximab	All vaccinations should be accomplished 4 weeks before treatment (special warnings in the SmPC), if necessary also shorter vaccination intervals (2 weeks) before the start of therapy are possible (off-label use).<br><br>For optimal success of the vaccination, at the earliest 6 months after the last administration of rituximab.<br>Influenza vaccination also during the therapy and within the 6-month interval after the end of therapy is recommended.<br><br>Safety at no time restricted.	Contraindicated during therapy.<br>Completion of immunization at least 4 weeks before the start of therapy, vaccinations at the earliest 12 months after therapy, but only after complete normalization of the B-cell count.<br>Infants who were in utero exposed to rituximab: Live vaccinations at the earliest after complete normalization of B-cell count.	Reduced but detectable immune response during therapy after influenza vaccination.<br>6 months after combination therapy with methotrexate decreased immune response after pneumococcus, but not after tetanus vaccination.

References

1. OCEBM Levels of Evidence Working Group “The Oxford Levels of Evidence 2”. Oxford Centre for Evidence-Based Medicine.

2. Brożek JL, Akl EA, Compalati E, et al (2011) Grading quality of evidence and strength of recommendations in clinical practice guidelines Part 3 of 3. The GRADE approach to developing recommendations. Allergy 66:588–595. https://doi.org/10.1111/j.1398-9995.2010.02530.x

3. Guyatt GH, Oxman AD, Kunz R, et al (2008) Going from evidence to recommendations. BMJ 336:1049–1051. https://doi.org/10.1136/bmj.39493.646875.AE

4. Wagner N, Assmus F, Arendt G, et al (2019) Impfen bei Immundefizienz : Anwendungshinweise zu den von der Ständigen Impfkommission empfohlenen Impfungen. (IV) Impfen bei Autoimmunkrankheiten, bei anderen chronisch-entzündlichen Erkrankungen und unter immunmodulatorischer Therapie. Bundesgesundheitsblatt Gesundheitsforschung Gesundheitsschutz 62:494–515. https://doi.org/10.1007/s00103-019-02905-1
